# Endemic Dysentery

**Published:** 1841-10

**Authors:** 


### THE WESTERN JOURNAL.

Vol. IV.—No. IV.

LOUISVILLE, OCTOBER 1, 1841.

### ENDEMIC DYSENTERY.

It seems that, in particular localities of the West, this disease has prevailed during the past summer, while large intervening districts have been nearly exempt. In the former it has often been fatal, especially to children. We hope our brethren where it prevailed, will favor us with histories of it for publication, which shall embrace an account of any thing in the condition of the earth, air, and water, which is not present every summer; together with notices of its pathological phenomena, and the treatment found by them to be most efficacious. D.

